# Pathogenic Impact of Fatty Acid-Binding Proteins in Parkinson’s Disease—Potential Biomarkers and Therapeutic Targets

**DOI:** 10.3390/ijms242317037

**Published:** 2023-12-01

**Authors:** Ichiro Kawahata, Kohji Fukunaga

**Affiliations:** 1 Department of CNS Drug Innovation, Graduate School of Pharmaceutical Sciences, Tohoku University, Sendai 980-8578, Japan; kfukunaga@tohoku.ac.jp; 2 BRI Pharma Inc., Sendai 982-0804, Japan

**Keywords:** Parkinson’s disease, tyrosine hydroxylase, dopaminergic neurons, fatty acid-binding protein, α-synuclein, mitochondria, dementia with Lewy bodies, biomarkers, therapeutic target, early diagnostic techniques

## Abstract

Parkinson’s disease is a neurodegenerative condition characterized by motor dysfunction resulting from the degeneration of dopamine-producing neurons in the midbrain. This dopamine deficiency gives rise to a spectrum of movement-related symptoms, including tremors, rigidity, and bradykinesia. While the precise etiology of Parkinson’s disease remains elusive, genetic mutations, protein aggregation, inflammatory processes, and oxidative stress are believed to contribute to its development. In this context, fatty acid-binding proteins (FABPs) in the central nervous system, FABP3, FABP5, and FABP7, impact α-synuclein aggregation, neurotoxicity, and neuroinflammation. These FABPs accumulate in mitochondria during neurodegeneration, disrupting their membrane potential and homeostasis. In particular, FABP3, abundant in nigrostriatal dopaminergic neurons, is responsible for α-synuclein propagation into neurons and intracellular accumulation, affecting the loss of mesencephalic tyrosine hydroxylase protein, a rate-limiting enzyme of dopamine biosynthesis. This review summarizes the characteristics of FABP family proteins and delves into the pathogenic significance of FABPs in the pathogenesis of Parkinson’s disease. Furthermore, it examines potential novel therapeutic targets and early diagnostic biomarkers for Parkinson’s disease and related neurodegenerative disorders.

## 1. Introduction

Parkinson’s disease is characterized by the progressive degeneration of dopaminergic neurons within the midbrain, particularly the nigrostriatal system, which plays a crucial role in motor function regulation. This degenerative process primarily occurs in the substantia nigra, leading to a substantial reduction in dopamine levels that significantly affects motor control, resulting in symptoms like tremors, muscle rigidity, and bradykinesia.

Despite extensive research efforts, the detailed etiology of Parkinson’s disease remains incompletely understood. The disease’s onset involves a complex interplay of genetic and environmental factors, including the abnormal accumulation and aggregation of α-synuclein, the pathogenic protein linked to Parkinson’s disease, as well as inflammatory responses and oxidative stress. While various studies have highlighted the impact of genetic mutations in familial Parkinson’s disease, the mechanisms triggering the disease in sporadic cases without clear genetic influences remain challenging to decipher.

Early therapeutic intervention is crucial for managing Parkinson’s disease; however, predicting its onset in the early stages remains challenging. Previous research indicates the potential utility of fatty acid-binding proteins (FABPs) as diagnostic biomarkers for various conditions, including cerebral infarction, Alzheimer’s disease, dementia with Lewy bodies, and Parkinson’s disease [1,2,3,4,5]. Thus, there remains a need for a deeper understanding of the molecular mechanisms underlying the involvement of FABPs in disease pathogenesis.

Therefore, this review aims to elucidate the mechanisms underlying the selective dysfunction of dopaminergic neurons and consequent neurodegeneration in Parkinson’s disease, focusing on the involvement of FABPs. Specifically, we highlight the emerging role of FABPs in α-synucleinopathies, presenting recent research elucidating novel mechanisms associated with α-synuclein propagation and toxicity through protein aggregation. Additionally, we delve into newfound insights into the molecular pathways governing mitochondrial dysfunction during the progression of neurodegeneration. Drawing from these research findings, we aim to explore the potential of novel pharmaceutical targets and biomarkers for effective disease prediction and therapeutic interventions.

## 2. Physiological Function of FABP and Involvement in Neurodegenerative Diseases

Fatty acids perform a variety of physiological functions in the body, serving as a source of energy for internal combustion, a major component of cell membranes, and a regulator of inflammatory responses. Excessive intake of fatty acids contributes to energy overload, obesity, and risk of brain inflammation. Fatty acids include saturated fatty acids; unsaturated fatty acids, which include monounsaturated and polyunsaturated fatty acids; and trans fatty acids. Some of these polyunsaturated fatty acids cannot be synthesized in the animal’s body and must be obtained from the diet. An excess of or deficiency in polyunsaturated fatty acids are associated with various functional disorders, neurological symptoms, and an increased risk of disease pathogenesis. Polyunsaturated fatty acids can be classified into omega-3 fatty acids, represented by docosahexaenoic acid (DHA) and eicosapentaenoic acid (EPA), and omega-6 fatty acids, which are metabolized into linoleic acid and arachidonic acid.

Because fatty acids are hydrophobic, they require carrier proteins to transport them to intracellular organelles, and FABPs are responsible for this physiological function [6,7,8,9]. FABP is a protein consisting of approximately 130 amino acid residues, and nine isoforms have been identified in humans [10,11]. Each of these isoforms is known to have some specificity in its expression distribution (Table 1). Three of these isoforms, FABP3, FABP5, and FABP7, are expressed in the nervous system [12,13,14,15]. FABP3, which was first identified in the heart [16,17], is abundantly expressed in mature neurons from the postnatal to the adult stage [14]. In contrast, FABP5 and FABP7 are maximally expressed in the fetus and neonate, during which time they are expressed in glial and neural stem cells [14,18,19]. FABP3 has a high affinity for n-6 polyunsaturated fatty acids such as arachidonic acid, FABP5 has a high affinity for saturated fatty acids such as stearic acid and palmitic acid, and FABP7 has a high affinity for n-3 polyunsaturated fatty acids such as DHA [20,21,22].

Polyunsaturated fatty acid itself affects the toxic expression of α-synuclein, the pathogenic protein of Parkinson’s disease. Previous reports indicate that polyunsaturated fatty acids bind to α-synuclein and promote oligomer formation [23,24,25]. The exposure of cultured mesencephalic neurons to polyunsaturated fatty acids increased α-synuclein oligomer levels [25]. In addition, when mice expressing A53T, a representative mutant family line of α-synuclein in familial Parkinson’s disease, were fed a DHA-containing diet, low concentrations of α-synuclein suppressed neuronal accumulation and toxic expression. In contrast, high concentrations of DHA increased intracellular accumulation of soluble and insoluble α-synuclein and neuronal injury [26]. In humans, fatty acids have also been implicated in the pathogenesis of Lewy body disease, and α-synuclein oligomers in the lipid fraction are detected in autopsy brains of Parkinson’s disease and Lewy body dementia patients but not in healthy subjects [25]. Furthermore, a higher intake of arachidonic acid, an omega-6 polyunsaturated fatty acid, has been suggested to increase the risk of Parkinson’s disease pathogenesis [27].

Furthermore, FABPs have pathogenic impacts and the potential for predictive biomarkers on various diseases, including brain injury and neurodegenerative disorders. FABP3 and FABP7 exhibit distinct distribution patterns within brain tissues, with FABP3 displaying notably higher concentrations in brain injury [28]. Elevated serum levels of FABP7 were observed in patients with Alzheimer’s disease, Parkinson’s disease, and other cognitive disorders [4]. In addition, FABP3 expression in the substantia nigra is known to be increased in autopsy brains of Parkinson’s disease patients [29]. In this context, FABP3 co-localizes with phosphorylated α-synuclein in Lewy bodies [30]. Serum FABP3 levels are increased in patients with Parkinson’s disease and dementia with Lewy bodies compared to healthy controls [3,31]. In addition, serum FABP3 was elevated in dementia with Lewy bodies and Parkinson’s disease with dementia, compared to non-dementia controls [2,32]. These clinical findings suggest that FABP3 may be involved in the pathogenesis of Lewy body diseases, including Parkinson’s disease. Based on these insights, the following chapters will describe the characteristics of Parkinson’s disease, the selective degeneration of dopaminergic neurons, and the pathogenic impact of FABPs in the disease.

**Table 1:** Tissue distribution and expressed cell types of the fatty acid-binding protein (FABP) subfamilies.

FABP Subfamily	Tissue Distribution	Expressed Cells	Ref
FABP1 (Liver FABP)	Liver, intestine, kidney, pancreas	Hepatocytes, enterocytes	[33,34,35]
FABP2 (Intestinal FABP)	Intestine	Enterocytes	[36,37,38]
FABP3 (Heart FABP)	Heart, skeletal muscle, brain	Cardiomyocytes, myocytes, neurons	[18,39,40,41,42,43]
FABP4 (Adipocyte FABP)	Adipose tissue, macrophages	Adipocytes, macrophages	[44,45,46,47]
FABP5 (Epidermal FABP)	Epidermis, brain, adipose tissue	Keratinocytes, adipocytes, glial cells, neurons	[18,43,48,49,50]
FABP6 (Ileal FABP)	Intestine	Enterocytes	[51,52,53,54]
FABP7 (Brain FABP)	Brain, eye, kidney, mammary gland	Neural stem cells, oligodendrocytes, astrocytes, ependymal cells	[18,41,43,55,56]
FABP8 (Myelin FABP)	Myelin-forming cells in the peripheral nervous system	Schwann cells, oligodendrocytes	[43,56]
FABP9 (Testis FABP)	Testis	Salivary gland, mammary gland	[57,58]

## 3. Pathology of Parkinson’s Disease and Current Issues

There are over 10 million Parkinson’s disease patients worldwide, accounting for 1–3% of the global population aged 60 and above [59]. Half of those aged 85 and above develop Parkinson’s disease [60]. The evolution of symptomatic therapies for Parkinson’s disease, including dopamine agonists as well as L-DOPA, has been remarkable [61]. However, a fundamental treatment for Parkinson’s disease has yet to be developed. Initiating treatment after onset does not lead to a favorable prognosis. At the onset of Parkinson’s disease, the pathogenic proteins have already accumulated in the brain, and this pathology of dopaminergic neuronal loss induces clinical symptoms. Therefore, there is an expectation for the establishment of methods to predict the onset early.

Parkinson’s disease was first diagnosed by James Parkinson in 1817, detailing its clinical features as tremor, rigidity, bradykinesia, gait disturbances, and postural instability [62]. Cognitive symptoms commonly emerge in the advanced stages of the disease [63]. Approximately 20–40% of all Parkinson’s cases develop Parkinson’s disease dementia, with an average progression time of 10 years. In total, 40% of Parkinson’s patients with severe olfactory dysfunction progress to dementia [64,65]. In this regard, the importance of the olfactory bulb as an entry site for prion-like transmission in neurodegenerative diseases is suggested [66]. Pathologically, Parkinson’s disease is characterized by the loss of dopamine-biosynthesizing neurons in the substantia nigra pars compacta. It is also denoted by the abnormal deposition of the pathogenic protein α-synuclein in the cell body and neuronal processes, forming Lewy bodies and Lewy neurites, respectively [67,68,69]. Parkinson’s disease, Parkinson’s disease dementia, and dementia with Lewy bodies share the accumulation and aggregation of the pathogenic protein α-synuclein within neurons, leading to the formation of Lewy bodies. Therefore, these Lewy body diseases are believed to share common pathological mechanisms.

The decline in motor function observed in Parkinson’s disease arises due to impaired nigrostriatal dopaminergic function [70]. Nigrostriatal dopaminergic projections centrally regulate voluntary movements, and their degeneration contributes to Parkinsonian clinical symptoms. In addition, the dopaminergic system, originating in the substantia nigra pars compacta and the ventral tegmental area, predominantly projecting to the striatum and prefrontal context, significantly influences behavioral activities [71,72,73]. Consequently, lesions in nigral neurons cause concurrent dysfunction of agonist and antagonist muscle pairs in animal models of Parkinsonism [74] and sporadic Parkinson’s disease [75]. The dopaminergic function is regulated by the neurotransmitter dopamine. This catecholamine is biosynthesized from L-tyrosine by the rate-limiting enzyme tyrosine hydroxylase (TH) and aromatic L-amino acid decarboxylase (AADC) [76]. TH requires tetrahydrobiopterin, which is biosynthesized by GTP cyclohydrolase I (GTPCH1), to perform its enzymatic activity [77,78]. Because the enzymatic activity of TH protein strictly controls the rate-limiting step of dopamine biosynthesis [79], unlike those of other dopamine biosynthesizing enzymes, the expression level and activity of TH, which is precisely regulated via phosphorylation, directly affect intracellular dopamine amount.

## 4. Biochemistry and Pathology of Tyrosine Hydroxylase

In the context of movement disorders, dopamine is an essential neurotransmitter for motor function [80]. In this context, TH is a rate-limiting enzyme for dopamine biosynthesis [79] and is selectively expressed in catecholaminergic neurons in the central nervous system. AADC is responsible for the subsequent reaction to TH [81,82], and GTPCH1 is essential for the biosynthesis of cofactors for TH [83,84], but their enzymatic activities are not strictly regulated. Therefore, TH activity is almost directly related to the amount of DA. Shortly, TH is a homotetramer consisting of four subunits through its C-terminal domain [85]. Its activity is primarily regulated via the phosphorylation of its serine 40 amino acid residue in the N-terminal region [86,87]. Phosphorylation of TH is facilitated mainly by the cAMP-dependent protein kinase (PKA), Ca^2+^/calmodulin-dependent protein kinase II (CaMKII), and also stress-responsive enzymes, including mitogen-activated protein kinase activated protein kinase (MAPKAPK) and mitogen- and stress-activated kinase 1 (MSK1) [87,88]. Phosphorylated TH is dephosphorylated by a protein phosphatase, such as protein phosphatase 2A (PP2A) [89].

**Figure 1:**
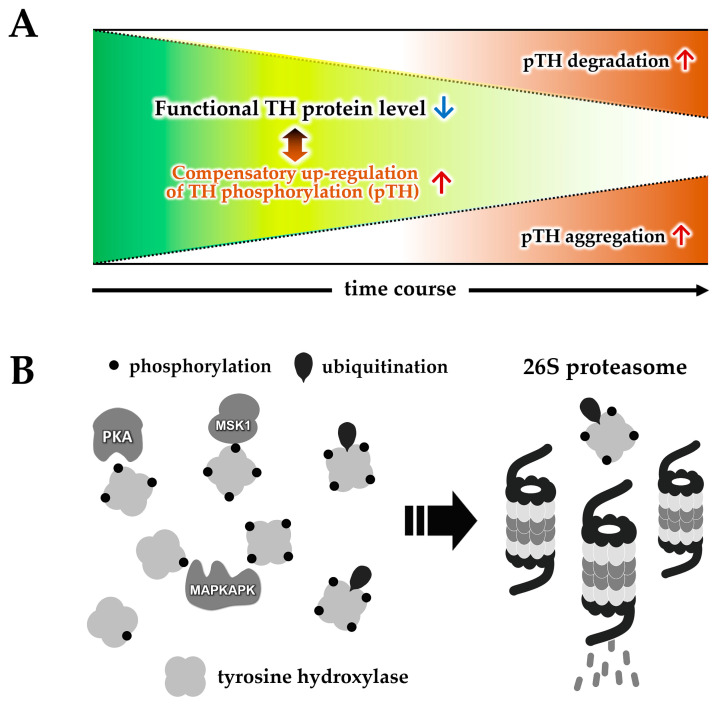
Degradation or aggregation of phosphorylated TH at serine 40 residue in the Parkinson’s disease model cells. (**A**) The dopamine-deficient state facilitates the phosphorylation of TH, which results in the degradation or aggregation of the protein [97,99]. (**B**) The schematic diagram of the TH degradation pathway in the Parkinson’s disease model cells [99,100,101]. pTH: TH phosphorylated at the serine 40; PKA: cAMP-dependent protein kinase; MSK1: mitogen- and stress-activated protein kinase 1; MAPKAPK: mitogen-activated protein kinase-activated protein kinase.

Dopamine-bound TH is inactive and stable, whereas TH is unstable in its phosphorylated active state [88,90,91]. The TH protein exhibits an aggregative nature upon phosphorylation [92,93]. In Parkinson’s disease, the levels of proteasome constituent proteins decrease, leading to reduced activity in the substantia nigra [94,95]. Furthermore, the oligomerization of α-synuclein, a pathogenic protein in Parkinson’s disease, impairs 26S proteasome-mediated protein degradation [96]. In this context, in Parkinson’s disease model cells treated with a proteasome inhibitor, the TH protein forms insoluble aggregates [97]. Specifically, phosphorylated TH at the serine 40 residue demonstrates an aggregation propensity but not dephosphorylated TH (Figure 1A). Indeed, immunohistochemistry reveals that Lewy bodies are immunopositive to TH phosphorylated at the serine 40 in the autopsied brains of Parkinson’s disease patients [98].

On the other hand, TH can be ubiquitinated [100], and the aggregates of phosphorylated TH are ubiquitin-positive [97]. In Parkinson’s disease and dopa-responsive dystonia, the TH protein in the substantia nigra striatum is lost. In model cells with reduced dopamine levels in Parkinson’s disease models or reduced biopterin levels in dopa-responsive dystonia, the phosphorylation level of TH increases, and over time, the TH level decreases [99]. This reduction in TH levels can be inhibited by a proteasome inhibitor. These findings suggest that the phosphorylation of TH is promoted by the decrease in dopamine or biopterin levels, and phosphorylated TH is likely degraded by the proteasome, explaining the mechanism of TH loss in Parkinson’s disease (Figure 1B).

## 5. Impact of FABP on the Loss of TH Protein and α-Synuclein Aggregation

As mentioned in the previous section, the activation of the TH protein by its phosphorylation accelerates its degradation in Parkinson’s disease model cells as well as the dopa-responsive dystonia model. In this context, the intracellular accumulation of α-synuclein promotes aggregation and phosphorylation, enhancing TH phosphorylation [102,103]. Intriguingly, the cellular uptake and accumulation of α-synuclein in dopaminergic neurons rely on FABP3 [104,105]. FABP3 is abundantly expressed in dopaminergic neurons within the central nervous system [18,42,43,104] and is immunopositive in intracellular inclusions in model cells [104] and in Lewy bodies in patients with Parkinson’s disease [30]. Indeed, in dopaminergic neurons in which FABP3 is knocked out, there is no increase in phosphorylated TH, and loss of TH is suppressed [101]. These data suggest that FABP3 affects the degradation and loss of TH protein through its involvement in α-synuclein aggregate formation and phosphorylation.

**Figure 2:**
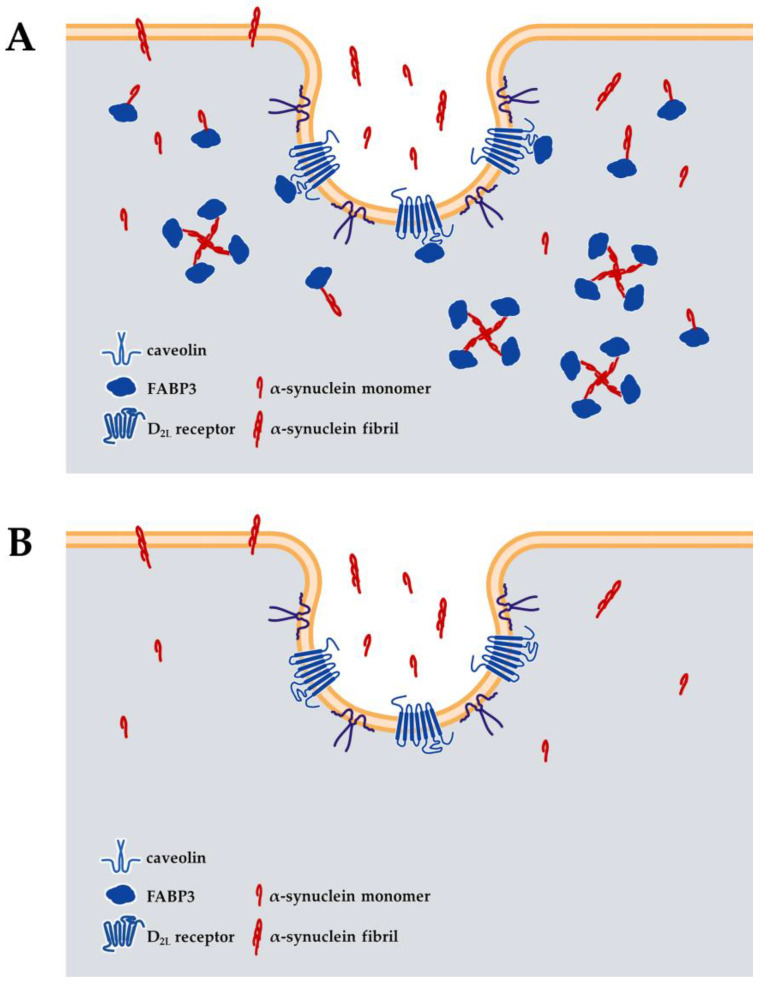
Schematic diagram of the involvement of FABP3 in the uptake and oligomerization of α-synuclein in neuronal cells. (**A**) FABP3 is scaffolded by dopamine D2 long type (D_2L_) receptors and is distributed near the plasma membrane, preferentially in the membrane rafts. FABP3 is critical for the α-synuclein accumulation and oligomerization to form aggregates [104,105,106,115]. (**B**) The absence of FABP3 abolishes α-synuclein accumulation and aggregation [104,105,106,107]. α-Synuclein that enters via the normal endocytic pathway is either degraded by endosomes or rapidly re-released via exocytosis. Note that there are various other mechanisms of α-synuclein uptake and possibilities for α-synuclein to enter the neuronal cells.

In the absence of FABP3, α-synuclein is not taken up by neuronal cells, thus preventing the formation of aggregates [104,105]. Additionally, FABP3 is not only involved in the intracellular uptake and aggregate formation of α-synuclein but also contributes to its propagation within the brain [106,107]. Furthermore, FABP3 is essential for the uptake of both monomeric and fibrillar forms of α-synuclein [105]. Its physiological function requires the presence of dopamine D2 receptors. There are two isoforms of dopamine D2 receptors: the long isoform of the dopamine D2 receptor (D_2L_ receptor) and the short isoform (D_2S_ receptor) [108]. The D_2L_ receptor has 29 more amino acids than D_2S_ and is located in the third intracellular loop. The D_2L_ receptor contains a binding site for FABP3, whereas the D_2S_ receptor lacks this binding site, indicating a selective interaction with FABP3 [109]. Analysis using D_2L_ receptor-specific knockout models suggests that the presence of both FABP3 and D_2L_ receptors is necessary for the uptake of α-synuclein [105]. Additionally, the abundance of D_2L_ receptors in the caveolae structures on the cell membrane is well documented [110,111], and the disruption of these structures leads to a decrease in the physiological function of FABP3 [105]. Based on this evidence, it is hypothesized that the D_2L_ receptor acts as a scaffold protein for FABP3, localizing it near the cell membrane and accelerating the intracellular accumulation of α-synuclein (Figure 2). The expression sites of FABP3 and D_2L_ receptors in the brain, such as the midbrain, olfactory bulb, and cerebral cortex, correspond to the regions where Lewy bodies are observed in Parkinson’s disease and Lewy body dementia [112,113,114]. This correspondence may provide an explanation for the site-selective formation of Lewy bodies in Lewy body diseases.

## 6. Pathogenic Impact of Fatty Acid-Binding Proteins on Mitochondrial Homeostasis

Mitochondrial dysfunction is critical for the pathogenesis of both familial and sporadic Parkinson’s disease [116]. 1-Methyl-4-phenyl-1,2,3,6-tetrahydropyridine (MPTP), a neurotoxin that originated as a byproduct of 1-methyl-4-phenyl-propionoxy-piperidine (MPPP) synthesis, is widely utilized in producing Parkinson’s disease models due to its induction of typical Parkinsonian symptoms effectively managed by L-DOPA [117]. Its ingestion results in pathological findings such as degeneration of substantia nigra dopaminergic neurons and the presence of Lewy bodies, as observed in postmortem brain analyses [118,119]. When MPTP enters the brain, it is oxidized by the enzyme monoamine oxidase B (MAO-B) within astrocytes and microglia to form 1-methyl-4-phenylpyridinium (MPP^+^), which is then released into the extracellular space and subsequently taken up by dopaminergic neurons. Once inside, MPP^+^ is internalized into the mitochondria, where it strongly inhibits complex I of the electron transport chain, leading to cellular degeneration due to decreased energy production capacity [120]. Rotenone [121], another well-known mitochondrial toxin, is also widely used to reproduce the Parkinson’s disease phenotype [122,123,124].

In this context, the knockout of the FABP3 gene or inhibition by FABP3-specific ligands ameliorates the axodendritic degeneration and neuronal loss induced by MPP^+^ [104] and MPTP [125,126]. Notably, the absence of FABP3 prevents the loss of mitochondrial membrane potential, abolishing the generation of stress response factors such as 4-hydroxynonenal (4-HNE) [104], which plays an essential role in the degeneration of dopaminergic neurons in Parkinson’s disease [127,128]. These observations suggest the pathogenic potential of FABP3 on the loss of mitochondrial homeostasis in Parkinson’s disease models. 

**Figure 3:**
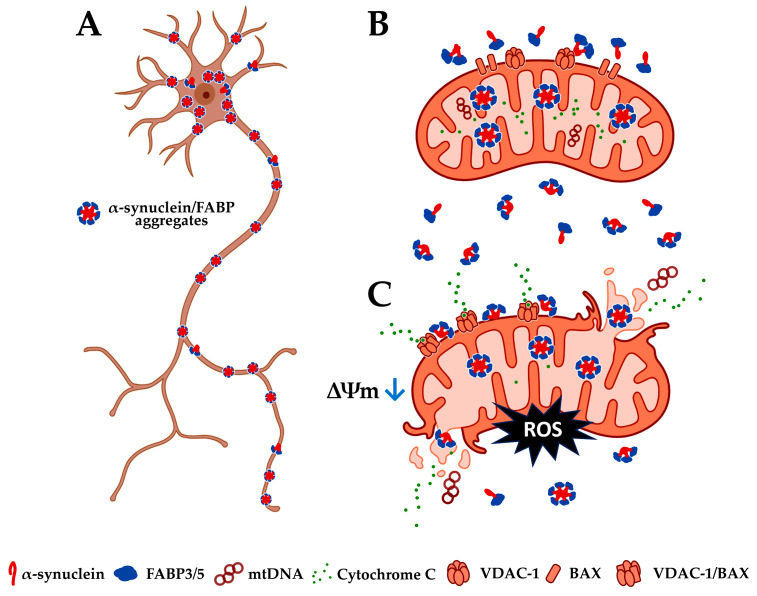
Schematic diagram of the involvement of FABP3 and FABP5 in mitochondrial dysfunction in neuronal cells of Lewy body disease model. Neurons express FABP3 and FABP5 in the process of neurodegeneration. (**A**) α-Synuclein is taken up into neurons and forms intracellular aggregations with FABP3 and FABP5 [104,105,107,129]. (**B**) Inflammatory responses induced by exposure to MPP^+^, rotenone, or transient cerebral ischemia cause α-synuclein and FABP3/5 to accumulate in mitochondria [104,129,132,133]. (**C**) FABP3 and FABP5 participate in mitochondrial VDAC-1/BAX complex formation to reduce intramitochondrial cytochrome C and membrane potential, resulting in apoptosis [130,133]. ΔΨm: mitochondrial membrane potential; VDAC-1: voltage-dependent anion-selective channel 1; BAX: Bcl-2-associated X protein; mtDNA: mitochondrial DNA.

Regarding other FABPs, FABP5 might also contribute to mitochondrial injury under rotenone-induced oxidative stress through its accumulation in mitochondria [129]. In addition, oligodendrocytes in Krabbe disease and the multiple sclerosis model show an abnormal localization of FABP5 in the mitochondria, leading to the formation of large pores in the mitochondrial outer membrane [130,131]. This pore formation triggers the release of inflammatory substances, leading to cell death. Furthermore, FABP3 and FABP5 are found together with 4-HNE in the mitochondria of neurons in the mice model of transient cerebral ischemia, which is also mediated by the formation of pores in the mitochondrial membrane [132,133]. These data suggest that not only FABP3 but also another subtype, FABP5, participates in mitochondrial dysfunction in the process of neuronal degeneration (Figure 3).

Glial cells are also closely involved in the pathogenesis of Parkinson’s disease [134,135]. FABP7 is involved in pathological processes in the central nervous system. Increased levels of FABP7 were observed in various parts of the hippocampus in response to cerebral ischemia in young adult monkeys [136,137]. In mouse models, FABP7 expression was up-regulated in glial fibrillary acidic protein (GFAP)-positive cells in response to spinal cord injury and autoimmune conditions [138], including experimental autoimmune encephalomyelitis [131,139]. Furthermore, FABP7 contributes to the accumulation of α-synuclein in mitochondria in oligodendrocytes to lose the membrane potential in the multiple system atrophy model mice [140,141]. These findings support the possibility of FABP7 participating in the process of neuronal degeneration.

## 7. Therapeutic Potential of FABP-Targeting Drugs for Parkinson’s Disease

As previously mentioned, the presence of FABPs is closely related to the maintenance of dopaminergic homeostasis and mitochondrial function in neurodegeneration. FABP3 and FABP5 are essential for the neurotoxicity expression of α-synuclein. Considering the potential for drug development targeting FABP3, we observed the absence of α-synuclein neurotoxicity in genetic knockout cells of FABP3. It was found that FABP3 can bind to α-synuclein in a 1:1 ratio, promoting α-synuclein-FABP3 aggregate formation [115]. Consequently, we attempted to create a drug that could inhibit the interaction between FABP3 and α-synuclein [126,142].

The FABP3 ligand, as a targeted drug for FABP3, inhibits α-synuclein aggregation and prevents its propagation in the brain and subsequent neuronal loss [106]. The FABP3 ligand effectively prevents the degeneration of dopaminergic neurons and restores motor dysfunction to a healthy level in a Parkinson’s disease mouse model [126]. Additionally, an α-synuclein mimicking peptide that inhibits the binding and aggregate formation of α-synuclein-FABP3 is capable of restoring memory and learning abilities in a Parkinson’s disease dementia mouse model [142]. This peptide has the potential to alleviate α-synuclein phosphorylation, which links to neurotoxicity. We are currently conducting preclinical trials of small molecules targeting FABP3 (Japan Agency for Medical Research and Development (AMED), Translational Research Program 23ym0126095h0002). Although the development of fundamental treatments for Parkinson’s disease is challenging, we aim to achieve early development and deliver it to Parkinson’s disease patients and their families.

With regard to FABP5, the specific ligand for FABP5 abolishes α-synuclein accumulation and translocation to mitochondria, which alleviates neurotoxicity in Parkinson’s disease model neuronal cells [129], as well as ameliorates oligodendrocyte injury in multiple sclerosis mouse models [131] and psychosine-induced apoptosis in Krabbe disease models [130]. Furthermore, the FABP5 ligand also has the ability to ameliorate FABP3/5-induced mitochondrial injury, including lipid peroxidation and BAX-related apoptotic signaling, indicating the potential neuroprotective treatment for ischemic stroke [132,133]. In addition, regarding FABP7, the ligand alleviates FABP7-induced α-synuclein oligomerization and aggregation, thereby rescuing glial and oligodendrocytes from cell death in multiple system atrophy model cells and mice [140,141,143]. These data suggest the promising potential of unique drug developments targeting FABP to regulate dopaminergic signaling and protect neuronal homeostasis.

## 8. Diagnostic Potential of FABPs as a Prodromal Biomarker for Parkinson’s Diseases

In recent years, there has been remarkable progress in targeted therapy for Parkinson’s disease, allowing for the precise alleviation of clinical symptoms [144,145]. However, challenges remain that affect the quality of life for patients, including subcutaneous device attachment, reduced medication efficacy due to disease progression, and the presence of OFF symptoms. In neurodegenerative diseases, post-onset treatment does not yield favorable prognoses, making fundamental treatment challenging. To overcome Parkinson’s disease, it is necessary to predict disease risk before onset and achieve early therapeutic interventions. Numerous excellent studies have been reported on diagnostic techniques for Parkinson’s disease using analyses of cerebrospinal fluid (CSF), serum, and plasma biomarkers targeting α-synuclein and other related proteins [146,147,148,149]. Additionally, recent studies have demonstrated that severe olfactory impairment is a useful early biomarker for Parkinson’s disease dementia [64,65,150,151,152].

Previously, certain reports have suggested methods to predict Parkinson’s disease using FABP3 as a biomarker in the cerebrospinal fluid or serum [2,32,153,154,155,156,157,158]. These investigations spotlight the pathogenic significance of the cerebrospinal fluid and serum FABP3 levels, indicating characteristic modifications in Parkinson’s disease, Parkinson’s disease dementia, and dementia with Lewy bodies. Elevated FABP3 levels are invariably observed in cerebrospinal fluid and serum specimens from patients with these disorders. Notably, higher FABP3 levels are observed in patients with dementia with Lewy bodies than in Parkinson’s disease dementia and Alzheimer’s disease, indicating the potential of FABP3 as a biomarker distinctive to dementia with Lewy bodies.

**Figure 4:**
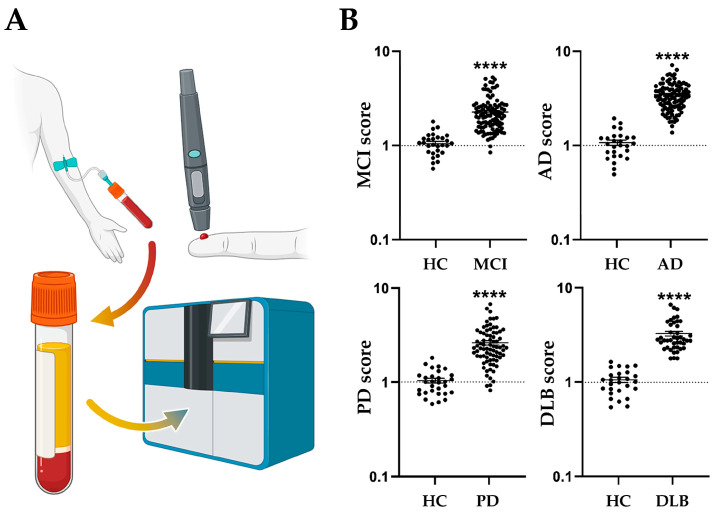
Technique for quantifying risk scores for neurodegenerative diseases using plasma biomarkers. (**A**) Flow chart from blood collection to plasma preparation and ELISA analysis. In addition to blood collection at the hospital, patients or healthy ones can collect their own blood at home. Plasma samples are analyzed using high-sensitivity immunoassays [31]. (**B**) Representative quantified data of risk scores for mild cognitive impairment (MCI), Alzheimer’s disease (AD), Parkinson’s disease (PD), and dementia with Lewy bodies (DLB) [31]. Student’s t-test is used to compare the means between each group. **** *p* < 0.0001 versus healthy controls (HC).

Therefore, with the aim of establishing a more accurate technique using plasma biomarkers to predict disease risk and discriminate potential disease types, we recently focused on FABP family proteins, essential for the propagation and expression of α-synuclein neurotoxicity, and developed a differential diagnostic technique for neurodegenerative disorders through a multi-marker analysis in combination with known biomarkers [31]. This method not only allows the discrimination between healthy individuals and those with Parkinson’s disease but also enables the prediction of Lewy body dementia, Alzheimer’s disease, and mild cognitive impairment. Specifically, we established a scoring technique that can differentiate Parkinson’s disease, Lewy body dementia, and Alzheimer’s disease from healthy individuals and also can distinguish between different neurodegenerative diseases (Figure 4) [31]. These multi-marker disease risk scores correlate with clinical symptoms in each disease, specifically mini-mental state examination (MMSE) scores and Hoehn–Yahr stages in Parkinson’s disease. We hope that this technology will contribute to the differentiation of clinically challenging conditions such as Parkinson’s disease dementia, Lewy body dementia, and Alzheimer’s disease in clinical settings.

In addition to ELISA analysis, metabolomics is a useful tool for measuring biomarkers [159,160]. ELISA analysis of plasma can measure the amount of a specific protein. This method is considered very accurate and reliable when the protein to be measured is known. Therefore, understanding the pathogenetic significance of the target proteins is essential. In contrast, metabolomics analysis, on the other hand, provides a comprehensive measurement of metabolites in vivo. The advantage of this method is that measurement is possible even if the metabolite to be measured is unknown. In this respect, compared to ELISA analysis, it may require more advanced techniques to account for measurement accuracy. Previous metabolomics studies successfully revealed lipid dysregulation in Parkinson’s disease [161,162]. Through utilizing the advantages of both of these approaches and establishing more accurate and highly specific biomarker predictions, it would be possible to produce a very early diagnosis of Parkinson’s disease before its onset.

## 9. Conclusions

In this review, we focused on FABPs and provided an overview of recent findings regarding the pathogenic impact of FABPs in the propagation and toxic expression of α-synuclein in neurodegenerative processes, as well as in the loss of mitochondrial homeostasis via VDAC-1/BAX complex formation to reduce intramitochondrial cytochrome C and membrane potential, resulting in apoptosis. FABP3 was found to be essential for the intracellular accumulation and oligomerization of α-synuclein, a pathogenic protein in Parkinson’s disease, as well as for its propagation in the brain. Additionally, FABP3 was responsible for the acceleration of TH phosphorylation and its subsequent degradation, which led to the loss of mesencephalic TH protein. FABP3 was also implicated in mitochondrial dysfunction and found to be a cause of ROS production in dopaminergic neurons.

In addition, FABP5 was also identified as essential for the toxic expression of α-synuclein through inducing apoptosis through the accumulation of α-synuclein in mitochondria and the formation of macropores on the mitochondrial membrane. Similarly, FABP7 was determined to be essential for the uptake and toxic expression of α-synuclein in glial cells, leading to apoptosis through its accumulation in mitochondria. Specific ligands for these FABPs were capable of inhibiting the formation of intracellular α-synuclein aggregates and mitochondrial injury, offering potential preventive therapeutic options for neurotoxicity.

Furthermore, the increased presence of FABP3 in the plasma of Parkinson’s disease patients indicated its potential benefit in predicting disease risk and discriminating from other α-synucleinopathies and Alzheimer’s disease, using multi-marker analyses. These findings suggest the utility of FABPs as pharmacological targets and biomarkers for Parkinson’s disease. We sincerely hope that these insights will deepen our understanding of the pathogenesis of Parkinson’s disease and contribute to the development of unique therapeutics and early diagnosis.

## 10. Patents

For the peptides used to prevent or treat synucleinopathy, the patent WO/2022/024693 can be referred to. Additionally, the biomarker for the diagnosis of dementia is documented in patent WO/2022/163818.

## Data Availability

Data are contained within the article.
